# *MLH1* Promoter Methylation Could Be the Second Hit in Lynch Syndrome Carcinogenesis

**DOI:** 10.3390/genes14112060

**Published:** 2023-11-09

**Authors:** Ileana Wanda Carnevali, Giulia Cini, Laura Libera, Nora Sahnane, Sofia Facchi, Alessandra Viel, Fausto Sessa, Maria Grazia Tibiletti

**Affiliations:** 1 UO Anatomia Patologica Ospedale di Circolo ASST-Settelaghi, 21100 Varese, Italy; nora.sahnane@asst-settelaghi.it (N.S.); fausto.sessa@uninsubria.it (F.S.); 2 Centro di Ricerca per lo Studio dei Tumori Eredo-Famigliari, Università dell’Insubria, 21100 Varese, Italy; laura.libera@uninsubria.it (L.L.); sofia.facchi@uninsubria.it (S.F.); mgtibiletti@gmail.com (M.G.T.); 3 Unit of Functional Oncogenomics and Genetics, Centro di Riferimento Oncologico di Aviano (CRO) IRCCS, 33081 Aviano, Italy; giulia.cini@asfo.sanita.fvg.it (G.C.); aviel@cro.it (A.V.); 4 Department of Medicine and Thecnological Innovation, Università dell’Insubria, 21100 Varese, Italy

**Keywords:** Lynch syndrome, *MLH1* methylation, constitutional epimutation, LS universal screening

## Abstract

(1) Background: *MLH1* hypermethylation is an epigenetic alteration in the tumorigenesis of colorectal cancer (CRC) and endometrial cancer (EC), causing gene silencing, and, as a consequence, microsatellite instability. Commonly, *MLH1* hypermethylation is considered a somatic and sporadic event in cancer, and its detection is recognized as a useful tool to distinguish sporadic from inherited conditions (such as, Lynch syndrome (LS)). However, *MLH1* hypermethylation has been described in rare cases of CRC and EC in LS patients. (2) Methods: A total of 61 cancers (31 CRCs, 27 ECs, 2 ovarian cancers, and 1 stomach cancer) from 56 patients referred to cancer genetic counselling were selected for loss of *MLH1* protein expression and microsatellite instability. All cases were investigated for *MLH1* promoter methylation and *MLH1/PMS2* germline variants. (3) Results: Somatic *MLH1* promoter hypermethylation was identified in 16.7% of CRC and in 40% of EC carriers of *MLH1* germline pathogenic variants. In two families, primary and secondary *MLH1* epimutations were demonstrated. (4) Conclusions: *MLH1* hypermethylation should not be exclusively considered as a sporadic cancer mechanism, as a non-negligible number of LS-related cancers are *MLH1* hypermethylated. Current flow charts for universal LS screening, which include *MLH1* methylation, should be applied, paying attention to a patient’s family and personal history.

## 1. Introduction

*MLH1* promoter methylation is a well-known epigenetic alteration in the tumorigenesis of colorectal cancer (CRC) and endometrial cancer (EC), the two most recurrent tumors found in Lynch syndrome (LS) patients. Lynch syndrome, also known as hereditary nonpolyposis colon cancer (HNPCC) syndrome, is an inherited syndrome (OMIM #120435, https://omim.org/entry/120435, accessed on 2 November 2023) responsible for about 3% of all colorectal cancers. It is caused by germline mutations in the mismatch repair (MMR) genes *MSH2, MSH6, MLH1,* and *PMS2* and is associated with high-level microsatellite instability (MSI-H) in tumor tissues. Patients with LS have an elevated risk of developing CRC and endometrial cancer (EC) (up to 80% and 60%, respectively) and are at a moderate risk for developing other types of cancer such as stomach, small bowel, bladder, other urothelial, and ovarian cancers [1]. Pathogenetic variants of the *MSH2* and *MLH1* genes account for the most part of LS, with a frequency of 40–60% and 40–50%, respectively, while minor percentages have been found for *MSH6* (10–20%) and *PMS2* (2%) [1]. Very recently, Mallorca Group suggested that MMR genes cause four dominantly inherited cancer syndromes with different penetrance and expressivity [2].

Carcinogenesis related to MMR defects is not an exclusive mechanism of LS; in fact, about 15% of sporadic CRC and 30% of sporadic EC are MMR-defective [3]. *MLH1* promoter hypermethylation is a well-known epigenetic alteration in the tumorigenesis of sporadic CRC and EC. In fact, non-inherited MMR-defective CRC is usually characterized by a *BRAF* V600E somatic mutation and/or *MLH1* promoter hypermethylation [4]. As well, non-inherited MMR-defective ECs are caused by *MLH1* somatic hypermethylation.

*MLH1* promoter hypermethylation causes gene silencing and, as a consequence, the loss of MLH1 protein expression and microsatellite instability (MSI) in tumor tissue. Usually, *MLH1* methylation is considered a somatic epigenetic mechanism that characterizes sporadic cancers, and it is used in the diagnostic flow chart based on universal tumor screening for mismatch repair defects to exclude LS [5,6]. In detail, when immunohistochemical (IHC) loss of MLH1 and PMS2 proteins is observed in a tumor, both *MLH1* germline variants and epigenetic silencing should be considered. *MLH1* promoter methylation and germline *MLH1* variants are commonly considered two mutually exclusive mechanisms in the carcinogenesis of CRC and EC [7,8]. In detail, when somatic *MLH1* hypermethylation and/or a *BRAF* mutation are identified in CRC, or only *MLH1* hypermethylation is identified in EC, patients are excluded from the LS germinal test, as reported by Tibiletti et al. [5].

Notably, the identification of patients and their relatives affected by LS is demonstrated by several studies as a cost-effective strategy for CRC and EC prevention [9]. It is well known that both intensive surveillance and risk-reducing surgery improve the long-term survival of LS patients [1]. For this purpose, *MLH1* promoter methylation and *BRAF* analyses are crucial to discriminate LS with respect to sporadic *MLH1*-defective CRCs and ECs. Nevertheless, *MLH1* hypermethylation has been described in rare cases of CRC and EC in LS patients who are carriers of pathogenic *MLH1* germline variants [10,11,12] or are carriers of a primary (de novo) or secondary (inherited) *MLH1* epimutation [13,14,15]. Primary epimutation corresponds to pure epigenetic events, and secondary epimutation corresponds to the secondary epigenetic effect of cis-acting genetic alterations transmitted following a Mendelian inheritance pattern [16].

The co-occurrence of *MLH1* methylation with germline pathogenetic variants has been recently highlighted by Moreira et al., who reported an important proportion, greater than 15%, of *MLH1* hypermethylated CRC from LS patients [17]. According to these data, somatic *MLH1* hypermethylation can be considered as a second hit of *MLH1* silencing in LS, and, in association with *MLH1* germline variants, as leading to the loss of function of MMR mechanisms and to the accumulation of errors during DNA replication.

Taking into account the pivotal role of *MLH1* methylation in the current flow charts for LS identification [5,8,18,19,20,21], in this study, a series of 61 cancers including 31 CRCs, 27 ECs, 2 ovarian cancers and 1 stomach cancer were studied in order to investigate the involvement of *MLH1* promoter hypermethylation in cancers of patients suspected for Lynch syndrome.

## 2. Patients and Methods

### 2.1. Patients Cohort

We retrospectively studied 61 cancers including 31 CRCs, 27 ECs, 2 ovarian cancers and 1 stomach cancer from 56 patients. As summarized in Table S1, the series included 41 women and 15 men and; the mean age of patients at diagnosis was 51.6 years (range 21–84 years). All patients were referred to the cancer genetic counselling service of ASST Sette Laghi in Varese from 2008 to 2020 to ascertain the presence of a cancer predisposition syndrome, according to the flow chart reported by the Italian Associazione Italiana Famigliarità ed Ereditarietà dei tumori Gastroenterici (AIFEG) consensus [1]. In detail, the investigated cases in this study were selected from all of the CRCs and the ECs diagnosed by the Department of Pathology at ASST Sette Laghi, and were subjected to the universal immunohistochemical analysis of the four MMR proteins and from cancers of patients referred to genetic counselling for suspected LS. Written informed consent was obtained prior to analysis for all patients who agreed to genetic testing.

The 61 cancers were selected for their absence of *MLH1* expression (*d-MLH1*) and, only for CRCs, *BRAF* wild-type status. All 61 cancers had been surgically removed and were evaluated by an expert pathologist (FS). As shown in the supplementary material (Table S1), the histological types of the series included 16 not otherwise specified adenocarcinomas (ADK), 13 mucinous (MUC), and 1 signet ring cell carcinoma (SRCC) for CRCs; 18 endometrioid, 1 papillary squamous cell carcinoma (PSCC), 1 squamous carcinoma (SCC), and 5 not otherwise specified adenocarcinomas (ADK) for ECs; 1 endometrioid ovarian cancer; and 1 intestinal type stomach cancer. Histological type was not available (na) for the remaining 4 cancers.

### 2.2. DNA Extraction and MSI Analysis

Tumor DNA was obtained from formalin-fixed and paraffin-embedded (FFPE) tissue using three representative 8 µm sections of tumor samples. In each section, neoplastic areas were selected by an expert pathologist (FS) and manually micro-dissected to minimize contamination by normal cells. DNA was extracted using a QIAamps DNA FFPE Tissue kit (Qiagen, Hilden, Germany) or a Maxwell^®^ DNA FFPE Kit and an automatized Maxwell 16 system (Promega, Madison, WI, USA), according to the manufacturers’ protocols.

MSI analysis was possible in 52 out of 61 cancers. The MSI analysis was performed on DNA using a pentaplex PCR panel of mononucleotide repeats (NR-21, NR-22, NR-24, BAT25, and BAT26), which are semi-monomorphic in the Caucasian population, as reported by Suraweera et al. [22]. The amplified fragments were subjected to electrophoresis using a SeqStudio genetic analyzer (Thermo Fisher scientific, Waltham, MA, USA) and were analyzed by two independent molecular biologists (GC and NS) using GeneMapper software version 5 (Thermo Fisher scientific, Waltham, MA, USA). Microsatellite instability was scored as a high level of instability (MSI-H) when at least 2 out of 5 of the analyzed microsatellites were unstable. A low level of microsatellite instability (MSI-L) was defined when only 1 microsatellite was unstable. When no microsatellite instability was identified for either locus, the sample was scored as microsatellite-stable (MSS).

### 2.3. MLH1 Methylation Analysis

The analysis of *MLH1* promoter methylation was performed using methylation-specific multiplex ligation-dependent probe amplification (MS-MLPA), using a SALSA MS-MLPA ME011 MMR kit (MRC-Holland, Amsterdam, The Netherlands) on the same tumoral DNA analyzed for MSI. MS-MLPA was performed according to the manufacturer’s instructions (www.mrc-holland.com, accessed on 2 November 2023). In brief, about 100 ng of DNA was hybridized for 16 h at 60 °C with methylation-specific probes, which contained an HhaI methylation-sensitive digestion site. The probes were subsequently digested with an HhaI enzyme, which digests only GCGC unmethylated sequences, and were amplified using PCR with universal FAM-labeled primers. PCR products were run on a SeqStudio genetic analyzer (Thermo Fisher scientific, Waltham, MA, USA) and checked with GeneMapper software version 5 (Thermo Fisher scientific, Waltham, MA, USA). Data analysis was carried out with Coffalyser.net software v.220513.1739 (MRC-Holland, Amsterdam, The Netherlands). According to the protocol, a sample was classified as methylated when CpG sites in the *MLH1* promoter region exhibited methylation with a level of methylation higher than a 0.2 ratio. This value corresponds to the limit of the blank for each probe. All data obtained through MS-MLPA analysis were confirmed by bisulfite pyrosequencing of the Deng-C region. In detail, *MLH1* bisulfite pyrosequencing addressed the methylation level of 5 CpG within the Deng-C region and was addressed using PCR amplification on bisulfite-converted DNA, followed by pyrosequencing (Qiagen, Hilden, Germany). A sample was classified as methylated when the mean of all five cytosines was higher than 10% of methylation. The 10% cut-off level was set by analyzing artificial control samples at different percentages of DNA methylation (0%, 10%, 50%, and 100%), which were prepared by mixing commercial fully methylated DNA and fully unmethylated DNA (Human WGA Methylated and Non-methylated DNA Set; Zymo Research).

Germline *MLH1* methylation analysis was performed on DNA extracted from blood using an MS-MLPA ME011 MMR assay (MRC-Holland, Amsterdam, The Netherlands).

### 2.4. Germline MMR Analysis

Germline analysis of the MMR genes including *MSH2*, *MSH6*, *MLH1*, *PMS2*, and *EPCAM* was performed on blood-derived DNA as per standard procedures using Sanger sequencing, targeted NGS panel sequencing, and MLPA testing, as previously reported [23]. In detail, this approach identified both point variants together with large deletions and duplications. The identified genetic variants were divided into five classes according to the International Agency for Research on Cancer recommendations and were classified in accordance with the guidelines from Insight Classification (InSiGHT Variant Interpretation Committee: Mismatch repair Gene Variant Classification Criteria, 2018; www.insight-group.org, accessed on 2 November 2023 [24]). Class 4 and 5 variants were considered pathogenic, while class 1 and 2 variants were considered benign and not reported. Class 3 variants were considered of uncertain clinical significance (VUS).

### 2.5. Statistical Analysis

Statistical analysis was performed using GraphPad Prism software (version 5, San Diego, CA, USA). Qualitative variables, such as presence or absence of *MLH1* methylation among cancer groups, were analyzed with the chi-square test with a 95% confidence interval (CI). *p*-values were considered significant when lower than 0.05.

## 3. Results

### 3.1. Somatic and Germline Analysis

Microsatellite instability (MSI) analysis was available for 52 out of 61 cancers. A high level of microsatellite instability (MSI-H) was identified in 96.1% (25/26) of the investigated CRCs and in 70.8% (17/24) of the ECs; only one EC revealed a low level of MSI (MSI-L). The two ovarian cancers revealed MSI-H patterns. MSI analysis was not possible for the stomach sample due to poor tumoral DNA quality.

Methylation analysis was performed on all samples and *MLH1* hypermethylation was observed in 25 out of 61 (41.0%) *d-MLH1* cancers, including 7/31 (22.6%) CRCs and 15/27 (55.5%) ECs. The two ovarian and one stomach cancers also revealed *MLH1* hypermethylation. Hypermethylated tumors showed a methylation level ranging from 0.2 to 0.6, with four cases (T-09 of patient P-09; T-10 and T-11 of patient P-10 from family F-5; T-43 of patient P-38 from family F-6) showing very high levels of methylation (greater than 0.9). For these four latter cases, *MLH1* methylation analysis was performed on blood DNA, and a constitutive *MLH1* hypermethylation was identified in each patient.

All patients affected by *d-MLH1* cancers were tested for the *MLH1* and *PMS2* genes in germinal setting, and pathogenic variants (class 4 and 5) of the *MLH1* gene were observed in 23 patients. Of these, five patients developed multiple cancers: P-10, P-13, P-16, and P-17 had both CRC and EC, while P-18 was affected by two CRCs. Overall, 28 tumors, respectively (18 CRCs and 10 ECs), belonging to 23 LS patients were considered. Only one patient (P-38 from Family F-6) affected by CRC carried the *PMS2* c.2380C>T p.(Pro794Ser) class 3 missense variant. Interestingly, the CRC of this patient showed a high level of *MLH1* methylation and the analysis of the blood sample demonstrated constitutional primary *MLH1* hypermethylation (supplementary Figure—Figure S1).

In Supplementary Table S1, we report all somatic and germline data for the 61 investigated cancers.

### 3.2. Correlation between Somatic Methylation and Germline Variants of MLH1 Gene

**Figure 1:**
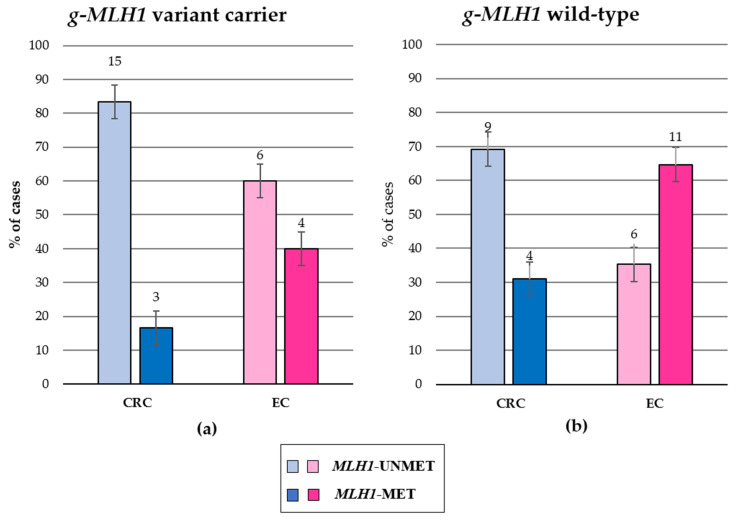
Distribution of samples according to *MLH1* germinal status (*g-MLH1*) and somatic *MLH1* promoter methylation in CRC (blue) and EC (pink). Lighter colors indicate the *MLH1* unmethylated samples, darker colors identify *MLH1* hypermethylated samples. (**a**) Somatic *MLH1* methylation status in CRC and EC associated with LS. (**b**) Somatic *MLH1* methylation status in sporadic CRC and EC. The bars indicate 95% confidence interval.

**Figure 2:**
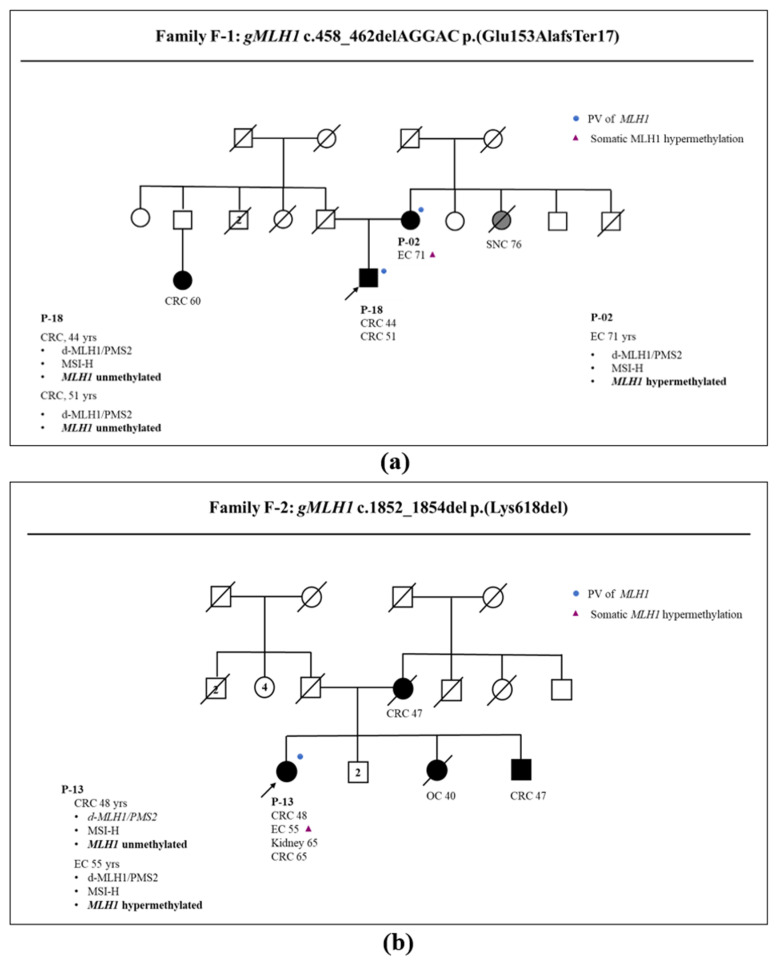
LS family pedigree: patient ID, cancer site, and age of onset are reported together with presence of *MLH1* germline pathogenic variants (blue dot) and somatic *MLH1* hypermethylation (purple triangle). Arrow indicates the proband. (**a**) Family F-1: patient P-18 is affected by two CRCs that are *MLH1* defective and negative for *MLH1* methylation P-02, mother of P-18, is affected by EC that is *MLH1* defective and showing *MLH1* hypermethylation; (**b**) Family F-2: patient P-13 is affected by CRC and EC; both tumors are *MLH1* defective but only EC revealed MLH1 hypermethylation. Legend: *gMLH1*: germinal *MLH1* status; PV: pathogenic variant; CRC: colorectal cancer; EC: endometrial cancer; d-MLH1/PMS2: defect of MLH1 and PMS2 protein expressions; MSH-H: presence of high level of microsatellite instability; IHC: immunohistochemistry.

**Figure 3:**
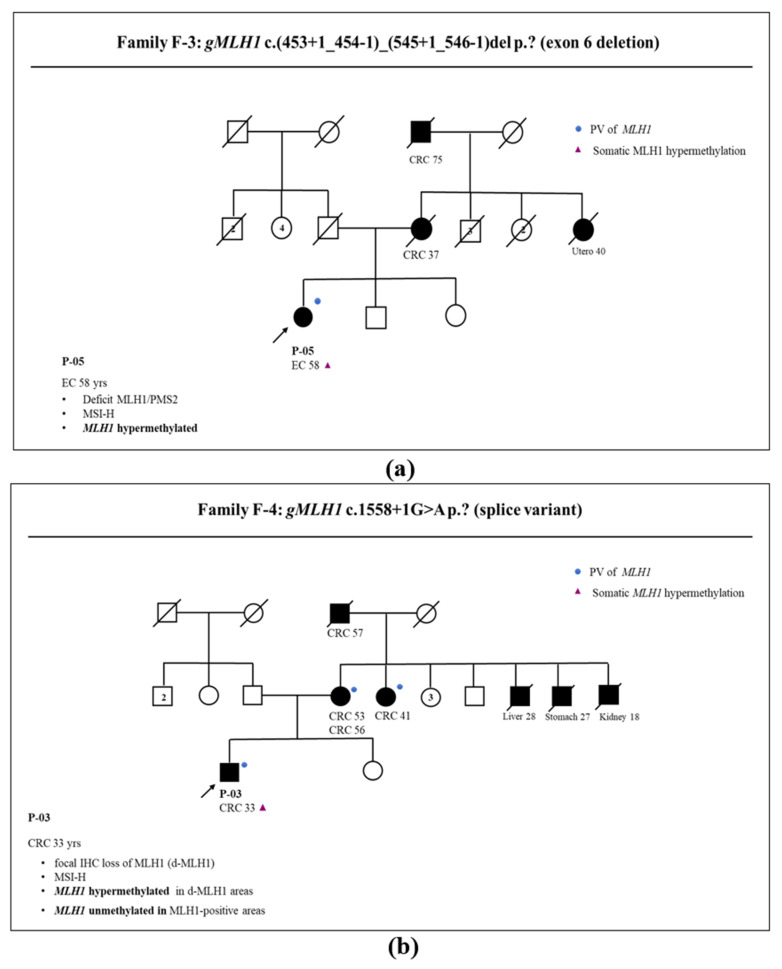
LS family pedigree: patient ID, cancer site, and age of onset are reported together with presence of *MLH1* germline (*gMLH1*) pathogenic variant (PV, blue dot). Arrow indicates the proband. (**a**) Family F-3: P-05 is affected by EC that is *MLH1* defective and demonstrates *MLH1* hypermethylation; (**b**) family F-4: patient P-03 is affected by CRC showing focal *MLH1* loss. In this tumor, *MLH1* hypermethylation was observed only in MLH1-defective areas. Legend: *gMLH1*: germinal *MLH1* status; PV: pathogenic variant; CRC: colorectal cancer; EC: endometrial cancer; d-MLH1/PMS2: defect of MLH1 and PMS2 protein expressions; MSH-H: presence of high level of microsatellite instability; IHC: immunohistochemistry.

**Figure 4:**
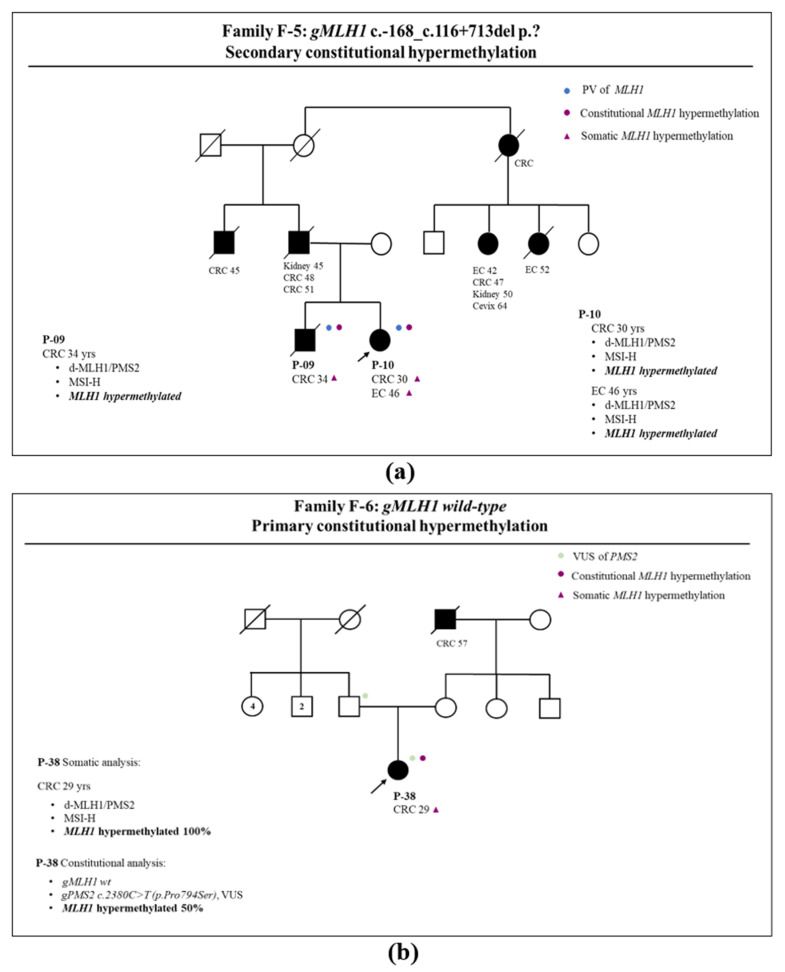
LS family pedigree: patient ID, cancer site, and age of onset are reported together with presence of *MLH1* germline variant (blue dot), *PMS2* germline variant (green dot), constitutional *MLH1* hypermethylation (purple dot), and somatic *MLH1* hypermethylation (purple triangle). Arrow indicates the proband. (**a**) Family F-5: patient P-09 is affected by CRC that is *MLH1* defective and *MLH1* hypermethylated; P-10 is affected by CRC and EC, with both tumors being *MLH1* defective and *MLH1* hypermethylated; (**b**) family F-6: patient P-38 is affected by CRC that is *MLH1* defective and *MLH1* hypermethylated. Legend: *gMLH1*: germinal *MLH1* status; *gPMS2*: germinal *PMS2* status; PV: pathogenic variant; CRC: colorectal cancer; EC: endometrial cancer; d-MLH1/PMS2: defect of MLH1 and PMS2 protein expression; MSH-H: presence of high level of microsatellite instability; VUS: variant of uncertain significance.

Figure 1 shows the distribution of somatic methylation in the 58 d-*MLH1* tumors from LS patients (*n* = 28, Figure 1a) and from sporadic patients (*n* = 30, Figure 1b). Remarkably, somatic *MLH1* promoter hypermethylation was identified in 3/18 (16.7%) CRCs and in 4/10 (40%) ECs of *MLH1* germline pathogenic variant carriers (Figure 1a). All seven cancers showing concomitant *MLH1* somatic hypermethylation and germline *MLH1* variants displayed immunohistochemical loss of expression of both *MLH1* and *PMS2*, presented with a high level of MSI, and belonged to five families (Figure 2, Figure 3 and Figure 4a). Although statistical analysis did not reveal a positive association between *MLH1* hypermethylation and LS (chi-square *p*-value = 0.0622), a relevant proportion of CRCs and ECs in LS patients showed *MLH1* hypermethylation.

Notably, different methylation patterns were observed in different cancers from the same family with the same constitutional *MLH1* pathogenic variant. In detail, as described in Figure 2a, two carriers of the *MLH1* c.458_462del p.(Glu153Alafs*17) variant from the same family (family F-1) had *MLH1* hypermethylated EC (P-02), while her son (P-18) had two *MLH1* unmethylated CRCs.

Analogously, patient P-13 (carrier of the *MLH1* c.1852_1854del p.(Lys618del) variant) from family F-2 (Figure 2b) developed multiple cancers, of which two were CRCs without methylation at the *MLH1* promoter and one was an *MLH1* hypermethylated EC. The different patterns of methylation in patients carrying the same germline variant suggest that somatic *MLH1* methylation is not linked to germinal condition in these patients, but rather that it occurs sporadically in cancer cells. Also, in family F-3 (Figure 3a), the concomitance of a germinal *MLH1* variant and somatic *MLH1* hypermethylation was ascertained in the index case (P-05). Overall, these constitutive and somatic conditions demonstrated that hypermethylation of *MLH1* could also occur sporadically as a second hit of an inherited pattern of *MLH1*. *MLH1* is a tumor suppressor gene, and it is well known that the loss of its function occurs as a consequence of a second hit involving *MLH1,* such as deletion, point mutation, or chromosome aneuploidy. Our data demonstrated that *MLH1* hypermethylation could also be a second hit occurring in LS cancers, causing loss of *MLH1* gene function.

Interestingly, in family F-4 (Figure 3b), the index case (P-03) carrier of the *MLH1* c.1558+1G>A pathogenic variant was affected by CRC, with clonal *MLH1* hypermethylation in those areas of tumors showing IHC clonal loss of *MLH1* expression.

On the contrary, in family F-5, three cancers (two CRC and one EC, Figure 4a) from two members (P-09 and P-10) carrying the c.168_c.116+713del *MLH1* variant revealed the same somatic methylation pattern. This family was previously described, and it was demonstrated that a large deletion of the promoter region of *MLH1* caused epigenetic silencing of this gene and was defined as secondary epimutation [23].

Finally, in family F-6 (Figure 4b), a young woman (P-38) who was a carrier of a variant of uncertain significance (VUS) in *PMS2* c.2380C>T p.(Pro794Ser), was affected by a CRC, with a high level of *MLH1* hypermethylation that was also confirmed in the blood sample. The presence of *MLH1* hypermethylation was also in the normal sample, which led us to define this condition as a constitutional *MLH1* hypermethylation (*MLH1* primary epimutation). Interestingly, three years later, this patient developed a complex MLH1-defective endometrial hyperplasia.

## 4. Discussion

It is well known that *MLH1* promoter methylation, together with the presence of a BRAF V600E somatic variant and a germline pathogenic *MLH1* variant, is generally considered to be a mutually exclusive mechanism in CRC carcinogenesis and that the epigenetic event is the principal mechanism of *MLH1* silencing in sporadic carcinogenesis [4,7,8]. On these bases, *MLH1* promoter methylation analysis is recommended in several guidelines and in universal CRC and EC screening in order to distinguish non-heritable from germinal origin of tumors [5,8,18,19,20,21]. However, the consolidated knowledge about these two mutually exclusive mechanisms was gained from the study of several CRC cohorts [7,8], whereas no data are available on ECs.

Recently, Yokoyama et al. [10] described an interesting case in which both an *MLH1* germline variant and *MLH1* promoter hypermethylation were observed in endometrial cancer in the same patient and concluded that LS cannot be excluded even if *MLH1* promoter hypermethylation on tumoral tissue is observed. The concomitance of the *MLH1* germline pathogenic variant and somatic epigenetic silencing of the *MLH1* gene was also described in very rare cases of constitutional primary and secondary epimutations [14]. Thus, differentiating “true sporadic” cases with somatic *MLH1* hypermethylation from seemingly sporadic cases with constitutional *MLH1* methylation or somatic methylation co-occurring with a deleterious germline *MLH1* variant poses a clinical and molecular diagnostic challenge.

In order to explore the relevance of the presence of *MLH1* promoter methylation in LS-related tumors, we investigated 61 cases including CRC and EC in relation to their *MLH1* germline condition. Interestingly, *MLH1* somatic hypermethylation was observed in concomitance with germline *MLH1* pathogenetic variants in CRC (16% of cases), in agreement with data reported by Moreira et al. [17], and more frequently in EC (40% of cases). The more common involvement of *MLH1* hypermethylation in EC with respect to CRC is highlighted in this work for the first time, and it is supported with evidence obtained from sporadic EC analyses [25,26]. In fact, as described by Borrego et al., in endometrial cancer, which is a hormone-sensitive tissue, non-genetic factors, such as body mass index and hormonal conditions, may play a role in the instauration of somatic *MLH1* methylation [25]. Nevertheless, a larger cohort of LS hypermethylated cancers is needed to validate these findings.

Pedigree analyses and methylation studies of the somatic and germinal status in the same family demonstrated that *MLH1* hypermethylation may represent a constitutional alteration co-occurring with a germline *in-cis* genetic variant (secondary epimutation, family F-5) or could be unrelated to any variants (P-38, family F-6). This latter case (P-38) showed a high level of *MLH1* methylation and was diagnosed as a primary epimutation, a germline condition of high cancer risk also in absence of Amsterdam criteria in this family [27]. Finally, *MLH1* methylation in LS-associated cancers could occur as the second hit in patients carrying a pathogenic germline *MLH1* variant. In fact, family F-2 showed that *MLH1* methylation is present only in EC and absent in both CRCs of the same patient (P-13). In family F-1, two carriers of the same *MLH1* germline variant, who were P-18 (the son) and P-02 (the mother), had two unmethylated CRCs (P-18) and a hypermethylated EC (P-02), respectively.

Moreover, even though our results were obtained from a very select cohort of patients, our findings are also confirmed by the recent literature [10,17], indicating that the *MLH1* epigenetic mechanism could be involved in LS carcinogenesis as a second hit. Altogether, the co-occurrence of *MLH1* hypermethylation and an *MLH1* germline variant suggests that *MLH1* hypermethylation should not be used to exclude LS.

Future studies are necessary to determine if the concomitant genetic and epigenetic *MLH1* inactivation is more frequent in EC in comparison with CRC and to understand if high levels of tumor *MLH1* promoter methylation may represent a real and useful marker of an underlying germline *MLH1* methylation.

## 5. Conclusions

In conclusion, our data confirmed that *MLH1* hypermethylation is not an exclusive mechanism of non-inherited cancers but also plays a non-negligible role in LS-related cancers, especially in EC. The co-occurrence of an *MLH1* germline mutation and somatic *MLH1* promoter hypermethylation was observed in different cancers of the same patient and in cancers of different patients from the same family. In these families, LS cannot be excluded even if *MLH1* promoter hypermethylation is observed. Thus, current flow-charts for universal LS screening, which include *MLH1* methylation testing to rule out LS, should be applied, also paying attention to a patient’s family and personal history even when somatic results are suggestive for sporadic cancer.

## Data Availability

Data are contained within the article and Supplementary Materials.

## Supplementary Materials

The following supporting information can be downloaded at: https://www.mdpi.com/article/10.3390/genes14112060/s1, Table S1: Comprehensive somatic and germline results of 61 *d-MLH1* cancers from 56 patients referred to cancer genetic counselling. Figure S1: ME011 *MLH1* methylation-specific multiplex ligation-dependent probe amplification (MS-MLPA) assay result of P-38 (family F-6). Top panel: Somatic analysis: methylation analysis output of the methylation-sensitive gene regions for *MSH2*, *MSH6*, *MLH1*, *MSH3*, *PMS2,* and *MGMT* genes (HHA1 site probes). Bottom panel: Constitutional analysis: methylation analysis output of the methylation-sensitive gene regions for *MSH2*, *MSH6*, *MLH1*, *MSH3*, *PMS2,* and *MGMT* genes (HHA1 site probes). The blue line indicates the cut-off level set at 0.2 of methylation (20%). In the black boxed area, the five probes for *MLH1* promoter methylation are highlighted. The figure shows full methylation of *MLH1* in the somatic setting and hemi-methylation in the constitutional panel.
