# Supplementary figures and images for: MLH1 Promoter Methylation Could Be the Second Hit in Lynch Syndrome Carcinogenesis
Source: Genes (Basel). 2023 Nov 9;14(11):2060. doi: 10.3390/genes14112060 (PMC10670941; doi:10.3390/genes14112060)

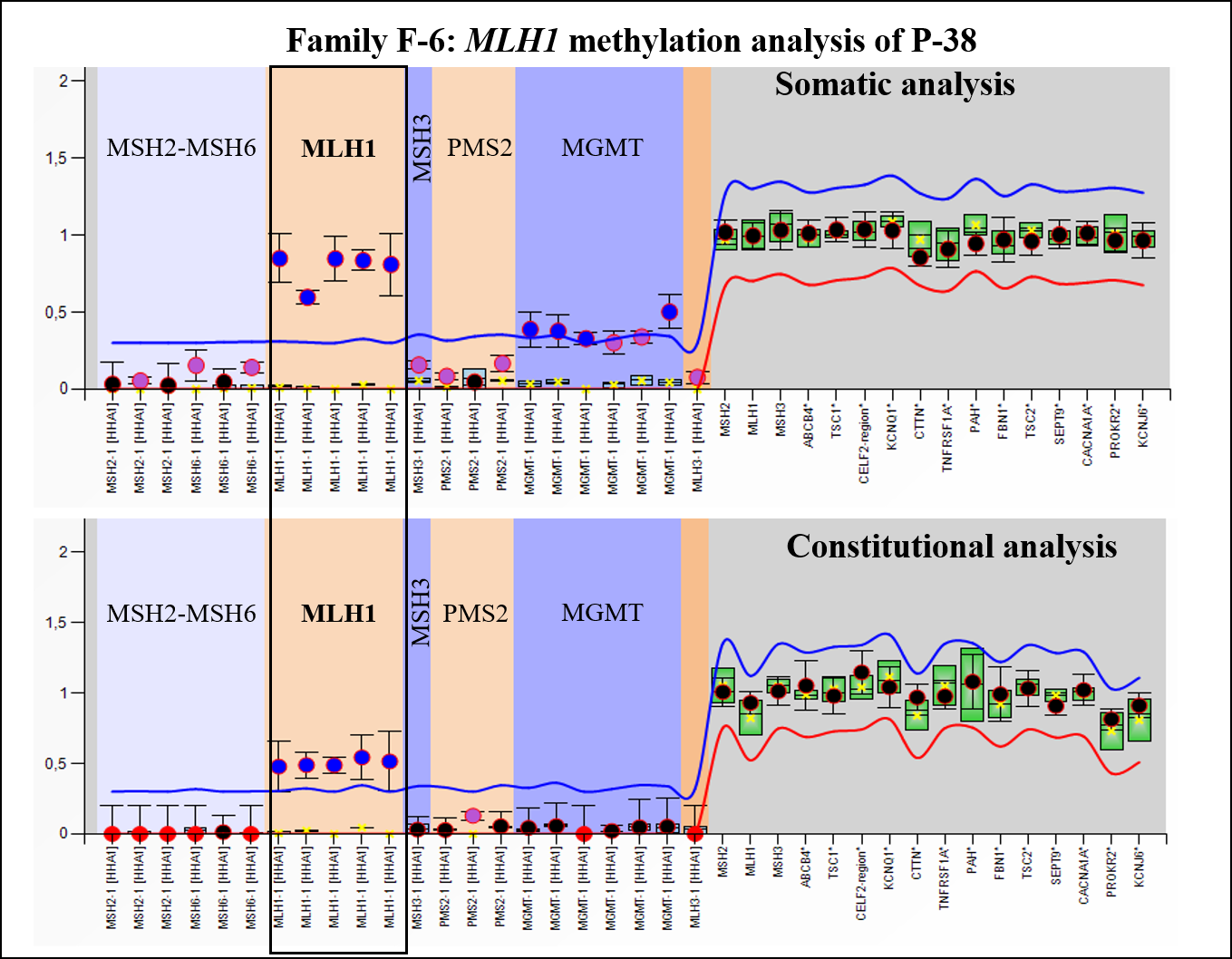

Supplement: Supplementary file 1 [file genes-14-02060-s001.zip › Figure S1.tif]
